# Congenital Aneurysm of the Muscular Interventricular Septum Associated With Bifascicular Block

**DOI:** 10.7759/cureus.29994

**Published:** 2022-10-06

**Authors:** Hoang H Nguyen, Patrick Y Jay

**Affiliations:** 1 Pediatrics, UT Southwestern Medical Center, Dallas, USA; 2 Pediatrics, Washington University School of Medicine, St. Louis, USA

**Keywords:** bifascicular block, vcg, vectorcardiogram, cardiac conduction defect, cardiac aneurysm, congenital heart defect

## Abstract

Isolated congenital aneurysm of the muscular interventricular septum is rare. We present a patient with congenital aneurysm of the basal muscular ventricular septum, who also develops conduction abnormalities with first-degree heart block, right bundle branch block, and left posterior fascicular block. The case details the natural history of the aneurysm over a 10-year period follow-up during which the patient remained asymptomatic with evidence of regression of the aneurysm. Given the aneurysm's location close to the proximal right bundle and left posterior fascicle, we believe that the cause for the aneurysm also injured both fascicles resulting in bifascicular block. The diagnosis of bifascicular block was confirmed using the electrocardiogram-derived vectorcardiography loops. These conduction abnormalities have remained stable. The case illustrates the utility of vectorcardiography in diagnosing bundle branch conduction defects. The case also illustrates the importance of anatomical considerations when encountering congenital heart defects.

## Introduction

Congenital malformations of the muscular interventricular septum are rare with only about 30-40 cases, a minority of which are familial, as described in the literature [1-3]. Patients are usually asymptomatic. We report a case of an aneurysm of the muscular interventricular septum associated with conduction abnormalities. First-degree atrioventricular block and bifascicular block affecting the right bundle and left posterior fascicle became manifest years after the aneurysm was diagnosed. The child remains well and asymptomatic after a decade of observation.

## Case presentation

**Figure 1:**
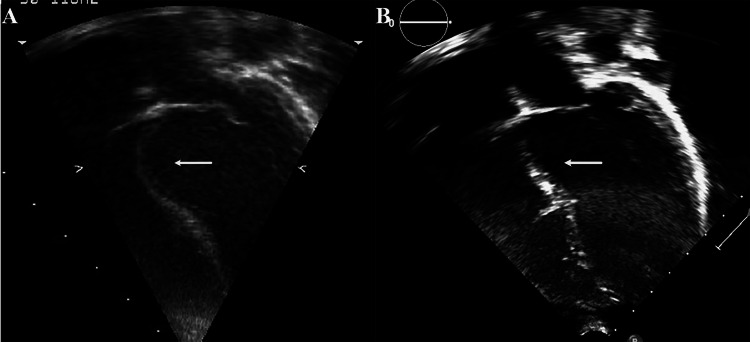
Echocardiogram images at presentation and last follow-up. A. Four-chamber view at end-diastole of the first echocardiogram at 18 months of age. There is an aneurysm of the basal interventricular septum at the level of the atrioventricular valves. Echogenic areas in the interventricular septum suggestive of ischemia/infarction secondary to an embolic event to the coronary arteries. B. Four-chamber view at end-diastole of the echocardiogram at last follow-up (12 years of age). The aneurysm is much less noticeable.

**Figure 2:**
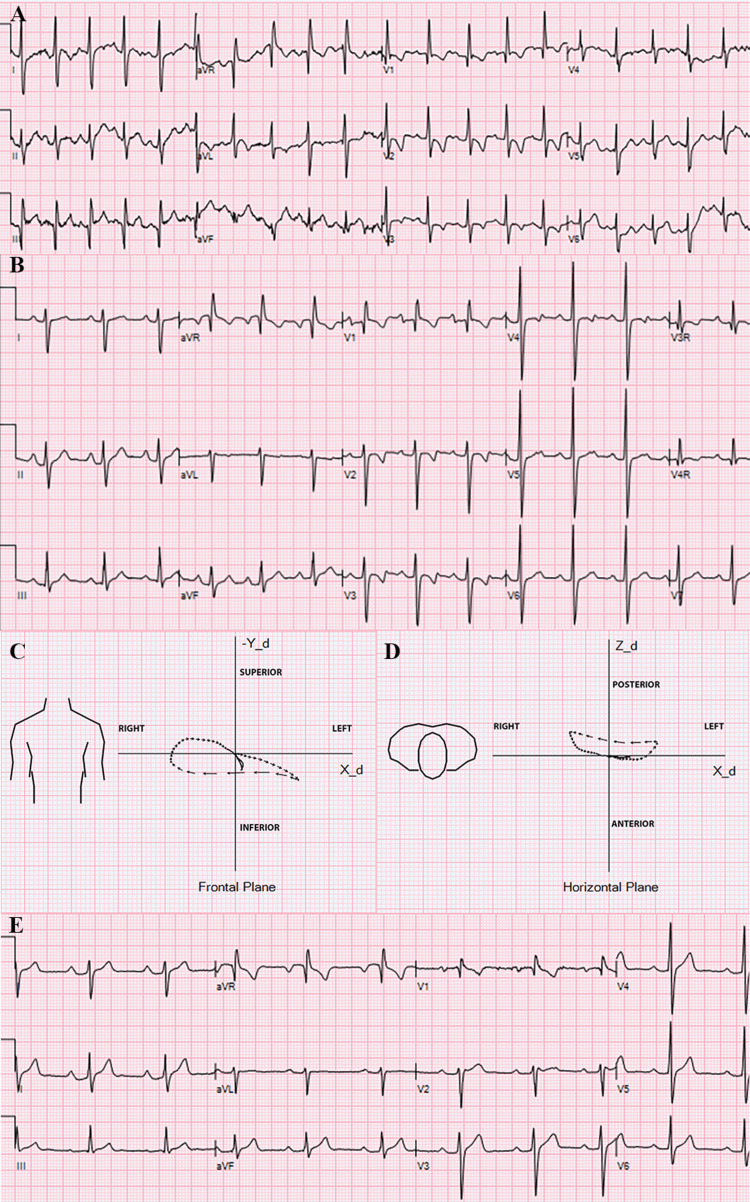
Electrocardiogram and vectorcardiogram at presentation and follow-up. A. 12-lead electrocardiogram at 18 months of age was normal for age. B. 12-lead electrocardiogram at 5 years of age when the conduction abnormalities (first-degree block, right bundle branch block, and left posterior block) are first seen. The PR interval is 200 msec. There is right axis deviation. The QRS duration is 110 msec. There is terminal R wave in V1 and the terminal S wave is wider than R wave in leads I and V6. C. Frontal plane loop of the derived vectorcardiography from the 12-lead electrocardiogram illustrative of the right bundle branch block and left posterior fascicular block. The initial vectors of the QRS loop are directed leftward. The vector inscriptions make a clockwise loop with terminal slowing directed rightward. D. Horizontal plane loop of the derived vectorcardiography of the 12-lead electrocardiogram illustrative of the right bundle branch block and left posterior fascicular block. The initial vectors are directed anteriorly and to the left. There is a counter-clockwise rotation of the QRS loop and rightward terminal slowing. E. 12-lead electrocardiogram at last follow-up at 9 years of age showing stable conduction defects. The PR interval is 200 msec, and the QRS duration is 110 msec.

A healthy, asymptomatic 18-month-old boy was referred for evaluation of an abnormal echocardiogram and innocent murmur. The echocardiogram showed an aneurysmal bulge of the ventricular septum just below the atrioventricular valves with mild hypokinesia of the septum (Figure 1A, Video 1). There were echogenic areas in the septum suggestive of ischemia/infarction due to an embolic event to the coronary arteries. The origins and appearance of the coronary arteries were normal. There was normal left ventricular size and function. No other defect was seen. The electrocardiogram showed sinus rhythm with an rsR’ pattern in V1. There was no ST segment or T wave abnormality (Figure 2A).

**Video 1:** Echocardiogram at presentation. Four-chamber echocardiographic view at presentation showing aneurysm of the interventricular septum and possible peeling of the endocardium.

The pregnancy and delivery of the child were normal. The past medical and family histories of the child and mother were unremarkable. Specifically, there was no history of hypercoagulability, congenital heart disease, cardiomyopathy, or cardiac conduction disease.

No further evaluation or intervention was recommended except periodic follow-up. The patient is currently 12 years old. He remains healthy and asymptomatic. The aneurysm became less noticeable on serial echocardiograms. The most recent echocardiogram showed slight thinning of the basal septum (Figure 1B, Video 2). The echogenic areas remain. The left ventricular dimensions and function were normal. No other defect or abnormality was seen. An electrocardiogram at five years of age showed first-degree atrioventricular block, right bundle branch block, and right axis deviation suggestive of left posterior fascicular block (Figure 2B). Vectorcardiogram loops derived from the surface 12-lead electrocardiograms support findings of bifascicular block (Figure 2C-D). Holter monitoring did not show worsening conduction delays at maximal heart rate of 174 bpm. These conduction defects have remained stable (Figure 2E).

**Video 2:** Echocardiogram at follow-up. Four-chamber echocardiographic view at last follow-up 10 years after presentation showing stable thinning of the basal interventricular septum.

## Discussion

Isolated congenital aneurysm of the basal interventricular septum is rare with little available data regarding its associated conditions and natural history. The majority of the congenital aneurysms of the basal interventricular septum are incidentally diagnosed on echocardiograms performed for other indications such as routine fetal ultrasound surveillance. While isolated aneurysms generally cause no symptoms, some have been associated with complications such as neonatal bundle branch blocks, development over time of dilated cardiomyopathy, outflow tract obstruction, aneurysm rupture, embolic stroke, and arrhythmias [1-3].

Given its location in the basal septum in proximity to the proximal right bundle and left posterior fascicle, we speculate that the original insult -- a small embolic injury predominantly to the base of the heart during fetal development -- that caused this aneurysm and echogenic foci in the septum also injured the left posterior fascicle resulting in bifascicular block. The presence of bifascicular block and first-degree heart block raises concerns for true trifascicular block. The conduction delay remained stable at a high heart rate, albeit not maximal heart predicted heart rate for age, on Holter monitoring suggesting that the PR prolongation is probably due to delay in the atrioventricular node. Given that he is asymptomatic, pacemaker therapy is not indicated at this time [4]. If he becomes symptomatic, a formal electrophysiology study is considered to locate the level of block and determine the need for pacemaker therapy. Coronary angiography or cardiac MRI may have shed light on the pathogenic mechanism, but neither was pursued because the child was well, and the studies were unlikely to affect clinical management. As the heart grew during childhood, the aneurysm may have become less apparent and the right and left posterior fascicular block more so due to the fixed size of the aneurysmal septum and conduction system.

## Conclusions

Depending on the location and size, aneurysm of the muscular interventricular septum could be associated with conduction defects. Aneurysm of the muscular interventricular septum could have a benign course and good long-term outcome. The resolution of the aneurysmal bulge and the stability of the conduction abnormalities over a decade of observation suggest that this child will likely remain asymptomatic. Nevertheless, follow up every three to five years was recommended.
